# Severe Phenotype of De Novo TSHR Activating Pathogenic Variants

**DOI:** 10.1155/crie/2254609

**Published:** 2025-09-30

**Authors:** Anna C. Torrey, Michael Zuccaro, Jonathan B. Steinman, Alex Guo, Helen Ellsworth, Michael A. Fremed, Vidhu V. Thaker

**Affiliations:** ^1^Vagelos College of Physicians and Surgeons, Columbia University, New York 10032, New York, USA; ^2^Division of Genetics and Genomics, Boston Children's Hospital, Boston 02115, Massachusetts, USA; ^3^Division of Pediatric Endocrinology, Department of Pediatrics, Columbia University Irving Medical Center, New York 10032, New York, USA; ^4^Division of Molecular Genetics, Department of Pediatrics, Columbia University Irving Medical Center, New York 10032, New York, USA; ^5^Department of Pathology, Columbia University Irving Medical Center, New York 10032, New York, USA; ^6^Division of Pediatric Cardiology, Department of Pediatrics, Columbia University Irving Medical Center, New York 10032, New York, USA

**Keywords:** aberrant recurrent laryngeal nerve, craniosynostosis, hyperthyroidism, TSHR activating variants

## Abstract

**Context:**

The phenotypic spectrum of thyroid-stimulating hormone (TSH) receptor (TSHR) pathogenic variants is broad. Germline variants causing constitutive TSHR activation in the absence of TSH result in familial nonautoimmune hyperthyroidism (FNAH) or sporadic nonautoimmune hyperthyroidism (SNAH). This hyperthyroid state, if present in utero or early childhood, can impact multisystem development. The consequences of severe early-onset hyperthyroidism have not been well described.

**Clinical Cases:**

Here, we report two unrelated individuals, each with a distinct monoallelic de novo TSHR pathogenic variant leading to severe congenital hyperthyroidism that required multistep thyroidectomies. Both patients had thyroid hypertrophy and vulnerable anatomic positioning of recurrent laryngeal nerves (RLNs), complicating surgical management. Case 1 is a 4-year-old boy with craniosynostosis and mitral valve dysplasia with SNAH caused by a heterozygous TSHR variant c.1515C >A; p.S505R. Hyperthyroidism was detected with thyroid storm at 17 months of age. Case 2 is a 9-year-old girl with SNAH and craniosynostosis from a novel heterozygous TSHR variant c.1897G >C; p.D633H identified in the neonatal period.

**Conclusion:**

The severe hyperthyroidism and complex course seen in these individuals contrast with previously reported cases. These cases highlight the wide phenotypic spectrum of TSHR activating variants and the persistent clinical sequelae of SNAH.

## 1. Introduction

Thyroid hormones play a critical role in metabolism, growth, and development across multiple organ systems [1]. Their circulating levels are tightly regulated by a feedback loop which involves hypothalamic thyrotropin releasing hormone (TRH) stimulating anterior pituitary release of thyroid-stimulating hormone (TSH). Hyperthyroidism caused by excessive thyroid hormone release can disrupt this homeostatic loop and lead to serious complications, including thyroid storm, craniosynostosis, and mitral valve disease .

TSH exerts its effects by binding to TSH receptor (TSHR), a G protein-coupled receptor located on the basolateral membrane of thyroid follicular cells. Ligand binding to the extracellular N-terminal domain of TSHR activates canonical signaling pathways involving cyclic AMP (cAMP), protein kinase A, and inositol 1,4,5-triphosphate cascades [2–10]. These pathways promote iodine uptake, thyroid hormone secretion, and thyroid cell proliferation [3–6, 11]. The resulting production of thyroxine (T4), which is peripherally converted to triiodothyronine (T3), maintains systemic thyroid hormone homeostasis.

Germline gain-of-function mutations in the TSHR cause familial nonautoimmune hyperthyroidism (FNAH), or sporadic nonautoimmune hyperthyroidism (SNAH) when arising de novo [12]. More commonly, hyperthyroidism is caused by stimulating autoantibodies against TSHR (Graves' disease), which mimic TSH and lead to receptor hyperactivation despite suppressed circulating TSH [13]. Similarly, pathogenic activating TSHR variants cause ligand-independent constitutive signaling, resulting in elevated intracellular cAMP and increased T4/T3 production [12]. Although efforts have been made to catalog pathogenic TSHR variants and assess their molecular and clinical impact [12, 14], genotype–phenotype correlations remain inconsistent. Here, we describe two unrelated patients harboring distinct de novo activating TSHR variants, each with a severe hyperthyroid phenotype.

## 2. Ethics Approval

This study was approved by the Columbia University Irving Medical Center Institutional Review Board (Protocol AAAR0351). Informed consent and assent were obtained prior to enrollment. Clinical data were obtained through direct communication with the family and extracted from electronic health records.

## 3. Case Presentation

### 3.1. Case 1

**Figure 1:**
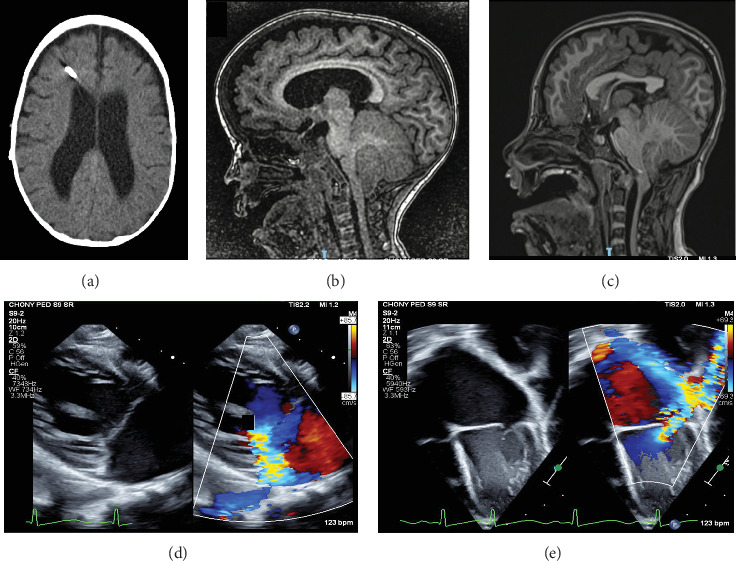
Imaging studies. (a) Case 1, axial CT imaging from 14 months of age showing ventricle dilation status postventriculoperitoneal shunt placement. (b) Case 1, Chiari I malformation with hydrocephalus, with cerebellar herniation and crowding of the foramen magnum at 1 year of age. (c) Case 2, Chiari II malformation with caudal displacement of the cerebellar tonsils, vermis and brainstem, elongation of the medulla, dysgenesis of the corpus callosum, and polymicrogyria at 8 years of age. (d, e) Case 1, preoperative echocardiogram images revealing severe mitral regurgitation in the long axis view (d) and apical view (e).

This 4-year-old male was born at 36 weeks' gestation and required a 7-day neonatal intensive care unit (NICU) stay for respiratory distress. Due to COVID-19–related disruptions in care, his first well visit occurred at 7 months of age, and he appeared developmentally appropriate at the time. By 9 months, macrocephaly (head circumference > 98th percentile) and developmental delay were noted. Brain imaging revealed Chiari malformation type I, sagittal suture craniosynostosis, and noncommunicating hydrocephalus of the lateral and third ventricles (Figure 1a,b). A ventriculoperitoneal (VP) shunt was scheduled and preoperative echocardiography revealed mitral valve regurgitation and left ventricle dilation, though no intervention was deemed necessary at that time (Figure 1d,e). Shunt placement at 10 months was complicated by acute respiratory distress and cardiac arrest secondary to prolonged hypoxemia. Viral PCRs revealed adeno-, rhino-, and enteroviral infection requiring a prolonged hospitalization. Furosemide was initiated to manage worsening mitral regurgitation.

At 17 months, he was readmitted with persistent tachycardia, diarrhea, and hypernatremia. Repeat echocardiography showed progression of mitral valve prolapse with leaflet thickening. He underwent mitral valve repair, including resection of abnormal papillary attachments and chordae, with partial improvement. However, postoperative complications included intermittent fevers, persistent tachycardia, and worsening residual mitral regurgitation, leading to thyroid function testing.

Thyroid function tests (TFTs) revealed thyroid storm: free T4 >77 pmol/L (ref. 10.3–24.5), total T3 8.97 nmol/L (ref. 1.2–2.4), and TSH <0.01 mlU/L (ref. 0.7–5.97). TSHR-stimulating antibodies were negative, suggesting SNAH. Trio whole exome sequencing (WES) was done using the Agilent SureSelectXT Human All Exon V5 + UTRs capture and sequenced on Illumina HiSeq2500. Reads were aligned to the human reference genome (GRCh37/hg19) and variants were annotated using NextGENe software from Softgenetics. Variants were prioritized and reported as described previously [15, 16]. This study identified a de novo heterozygous pathogenic variant in TSHR (c.1515C >A; p. Ser505Arg; alpha missense score = 0.996 [likely pathogenic], REVEL score 0.70 [damaging, likely pathogenic]) affecting the third transmembrane domain of the TSHR, a known activation-sensitive domain. Two other pertinent variants of uncertain significance (VUS) reported in WES were ruled out based on ACMG guidelines [17]. These included *SMAD3*, related to craniosynostosis, shared with unaffected mother, and *DCHS1*, related to mitral valve prolapse, inherited from unaffected father, both not deemed causal.

**Figure 2:**
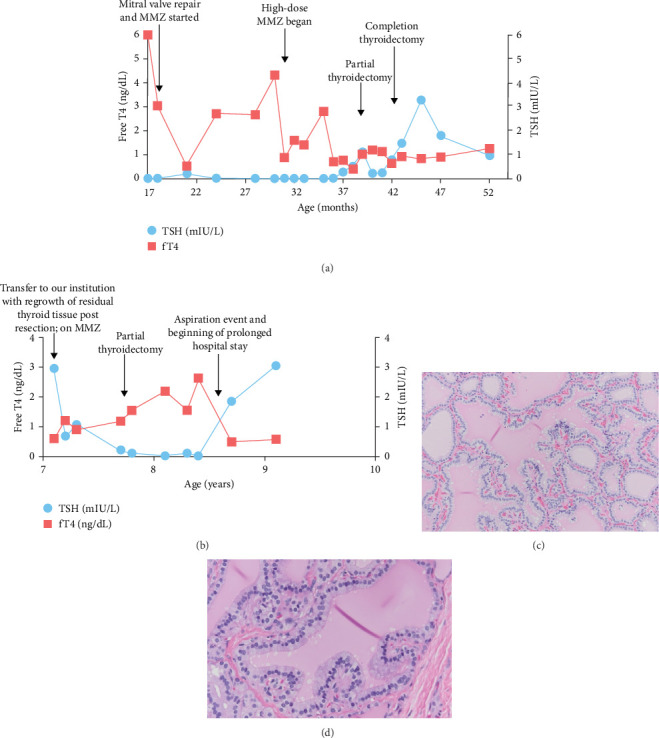
Thyroid function status and pathology. (a) Serum TSH and free T4 (fT4) concentrations of case 1 with methimazole (MMZ) treatment from diagnosis at 17 months of age to current age of 52 months. (b) Serum TSH and fT4 concentrations of case 2 from transfer to our institution at 7 years of age through current age of 9 years. (c, d) Thyroid follicles show columnar epithelium and basally oriented nuclei with epithelial infolding, consistent with follicular hyperplasia, which is present in both native thyroid tissue from patient 1 (Figure 2c, 20x) and residual, regrown tissue from patient 2 (Figure 2d, 40x). The colloid is moderately eosinophilic to pale pink with scalloping at the luminal aspect of epithelial cells.

Initial treatment of thyroid storm included methimazole, propranolol, potassium iodide, and hydrocortisone. While maintenance with methimazole continued, he had poor weight gain, feeding intolerance, and tachycardia, necessitating gastrostomy tube (G-tube) placement. He also developed diffuse goiter by 18 months and obstructive sleep apnea and continued treatment with furosemide for chronic mitral regurgitation. Due to persistent thyroid hormone dysregulation (Figure 2a), a block-and-replace strategy was adopted using high-dose methimazole with exogenous levothyroxine supplementation, which resulted in improved weight gain and growth velocity. Close monitoring and medication adjustment allowed for achievement of euthyroid state in advance of thyroid surgery.

At 39 months, a planned total thyroidectomy, by a highly experienced surgeon, was converted to partial procedure due to loss of the left recurrent laryngeal nerve (RLN) signal intraoperatively. RLN recovered, and completion thyroidectomy was successfully performed at 42 months. Pathology examination confirmed diffuse follicular hyperplasia (Figure 2c). Notably, the RLN were found to be medially displaced and stretched, consistent with the distortion of normal anatomy due to thyroid hypertrophy. The second surgery was complicated by the transection of the right RLN, which could not be reanastamosed. Postoperative monitoring confirmed right vocal cord paresis, with continued surveillance for aspiration risk.

Thyroid function was fully normalized postoperatively on daily thyroid hormone replacement.

At 4 years of age, he was weaned off the G-tube feeds, demonstrated appropriate linear growth (height 61st percentile and weight 39th percentile), and showed steady gains in developmental milestones. At age 5, he is able to say numbers, words, and colors, demonstrates age-appropriate mother and adaptive skills, including walking up and down stairs without support and drawing lines and circular scribbles. He continues to tolerate the residual moderate-to-severe mitral valve regurgitation with daily furosemide. Future mitral valve replacement is anticipated.

### 3.2. Case 2

This 9-year-old female was born at 31 weeks' gestation via C-section due to placenta previa and pre-eclampsia. She required intubation for 2 days and had a 5-week NICU stay for prematurity-associated complications, including persistent tachycardia. TFTs revealed free T4 of 103.2 pmol/L (ref. 11.5–28.3), TSH of 0.012 mlU/L (ref. 0.7–11.0), and negative TSHR antibodies, consistent with nonautoimmune hyperthyroidism. Targeted genetic testing for SNAH identified a heterozygous pathogenic TSHR variant (C0.1897G >C; p. Asp633His; alpha missense score 0.983 [likely pathogenic]; REVEL score 0.58 [damaging]), located in the transmembrane helix 6 (TM6). This residue is critical for maintaining receptor inactivation; substitution to histidine destabilizes the inactive state and leads to constitutive receptor activation. The variant was confirmed to be de novo through parental genetic testing. Methimazole was initiated for thyroid hormone suppression.

At 3 months, her mother noticed frontal bossing and proptosis. At 8 months, the head CT showed left coronal craniosynostosis, Chiari malformation type II, and severe obstructive hydrocephalus with transependymal edema (Figure 1c). A VP shunt was placed, and the proptosis was attributed to craniofacial dysmorphology and not autoimmune orbitopathy. At 12 months, she exhibited global developmental delays and severe failure to thrive, with weight and length below the 1st percentile, using the WHO child growth standards (girls, birth to 2 years) [18].

Despite methimazole therapy, hyperthyroidism remained difficult to control, and she developed severe OSA by 4 years of age with goiter on exam. She underwent adenotonsillectomy and a planned complete thyroidectomy. Planned total thyroidectomy was complicated by intraoperative injury to the right RLN necessitating an incomplete resection. Surgical findings suggested medial deviation and increased tension on the RLN, likely reflecting a distorted nerve course caused by an enlarged thyroid gland. She also developed postoperative hypoparathyroidism, requiring calcitriol and calcium supplementation.

Subsequent growth of the residual thyroid tissue resulted in the development of two 18 mm cystic nodules in the thyroid bed. These were aspirated and partially resected at 7 years of age. Pathologic examination showed multinodular tissue with papillary hyperplastic change (Figure 2d), without malignancy. The remaining thyroid remnant continues to enlarge causing tracheoesophageal deviation. Despite this, her thyroid function is within the normal range on methimazole (Figure 2b).

**Figure 3:**
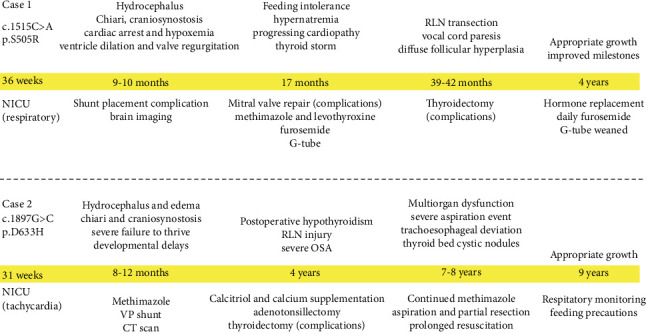
Clinical course summary for cases with de novo pathogenic TSHR variants. This timeline summarizes the major clinical events and interventions for two individuals harboring distinct pathogenic variants in a thyroid hormone–related gene: Case 1 (c.1515C >A, p.S505R) and Case 2 (c.1897G >C, p.D633H). Both cases presented with early-onset multisystem disease, including hydrocephalus, Chiari malformation with craniosynostosis, and severe cardiac and endocrine manifestations. Key time points are highlighted in yellow and represent major clinical inflection points, including neonatal presentation, critical surgical interventions, and longitudinal follow-up through 4 and 9 years of age, respectively. G-tube, gastrostomy tube; NICU, neonatal intensive care unit; OSA, obstructive sleep apnea; RLN, recurrent laryngeal nerve; VP shunt, ventriculoperitoneal shunt.

At 8 years of age, she experienced a severe aspiration event requiring prolonged resuscitation and intensive care. She suffered multiorgan dysfunction and neuropathic injury, resulting in right foot drop and right-hand ischemia. She continues to require feeding precautions and respiratory monitoring due to aspiration risk. As of age 9, she is growing appropriately in height and weight, and the VP shunt remains functional. Both cases are summarized in Figure 3.

## 4. Discussion

This report describes two unrelated children with de novo activating TSHR pathogenic variants causing severe SNAH. Activating TSHR pathogenic variants exhibit variable expressivity across familial and sporadic cases [10, 14]. In our cases, the clinical severity—marked by diffuse thyroid hyperplasia, craniosynostosis, hydrocephalus, Chiari malformations, and mitral valve dysplasia—far exceeds the typical phenotypic range of FNAH. De novo variants, lacking evolutionary filtering, are often more deleterious than inherited variants, possibly explaining the observed extreme phenotype [19]. Importantly, both children likely experienced in utero thyroid dysfunction, emphasizing the role of early diagnosis and treatment in mitigating developmental complications.

Both cases fulfilled criteria for clinical SNAH [12], with elevated free T4 and T3, suppressed TSH, negative thyroid autoantibodies, and genetic confirmation of pathogenic TSHR variants. Case 1 was diagnosed during a thyroid storm at 17 months; Case 2 in the neonatal period. Despite initial control with methimazole, both required thyroidectomy due to persistently uncontrolled hyperthyroidism and thyroid gland hypertrophy. The extent of thyroid hyperplasia in both cases was highlighted by the vulnerable position of the RLNs, resulting in injury despite intraoperative monitoring by an experienced high-volume surgeon [20]. Intraoperative findings indicated that the RLNs were medially displaced and stretched, likely due to mass effect and anatomical remodeling caused by the hypertrophic thyroid glands, further complicating nerve preservation during resection. While thyroid hypertrophy and thyrotoxicosis have been described in FNAH caused by a TSHR p.Ser505Arg [21–25] variant, these two cases represent more severe and earlier manifestations, likely reflecting mutation context and allele origin.

One striking feature was the presence of craniosynostosis in both patients. This likely reflects a combination of excess circulating thyroid hormone and TSHR-mediated skeletal signaling. Thyroid hormones have a well-established role in the growth and maintenance of the developing and adult skeleton [26]. Thyrotoxicosis accelerates rapid bone turnover and can induce premature suture closure in infants [27–29], supported by murine studies demonstrating T3-driven osteogenesis and expedited suture fusion [30]. Furthermore, activated TSHR may play a direct role in osteogenesis at the site of bone. TSHR is expressed in osteoblasts, osteoclasts, and chondrocytes and participates in skeletal remodeling [31]. Constitutive TSHR activation mimics chronic TSH stimulation, enhancing osteoblast activity and suppressing osteoclastogenesis, promoting rapid bone formation [32–34]. While craniosynostosis has previously been reported with some TSHR variants [35–37], it has not previously been associated with the S505R or D633H, underscoring the severity and novelty of these presentations.

Craniosynostosis may have contributed to the hydrocephalus and Chiari malformations observed in both cases. Premature suture fusion can distort cranial architecture and increase intracranial pressure, compressing developing brain structures and altering cerebrospinal fluid dynamics [38]. These findings support the hypothesis that TSHR overactivation can impact neural development indirectly through skeletal remodeling.

Case 1 also presented with mitral valve prolapse and severe regurgitation, likely related to both elevated thyroid hormone and direct cardiac TSHR signaling. TSHR is expressed in cardiomyocytes, where its activation increases intracellular cAMP signaling and may contribute to cardiac hypertrophy [39–41]. While mitral valve prolapse has been reported in one family with a TSHR c.2016C >G p.Cys672Trp activating variant [21, 22, 36], our case provides further evidence of valvulopathy as a potential consequence of early-onset TSHR overactivation. The complexity of the cardiac anatomy and mitral valve repair, and continued dysfunction despite surgery, underscore the long-term developmental impact on cardiac structure.

The TSHR p.Ser505Arg variant identified in Case 1 has been previously implicated in sporadic and familial forms of NAH [21, 22, 42, 43]. Functional studies using transfected COS-7 cells demonstrated that this substitution results in increased basal cAMP production, consistent with ligand-independent constitutive receptor activation [23]. Structurally, Ser505 lies within the transmembrane helix 3 (TM3) of the receptor. Substitution with a positively charged arginine is predicted to disrupt interhelical packing, particularly with TM6 and TM7, and favoring a stabilized active receptor conformation [44]. Clinically, affected individuals typically presented in early infancy with tachycardia, poor weight gain, hyperactivity, and were found to have suppressed TSH and elevated T4/T3 levels in the absence of thyroid autoantibodies. Additional features often included goiter and advanced bone age. Some cases were managed with antithyroid medications, while others required thyroidectomy for definitive controls. In several reports, the parents were identified to carry the variant through cascade genetic testing. In one family, a child was diagnosed at 6 months due to poor weight gain and found to carry the p.Ser505Arg variant. The same variant was subsequently identified in the father, who had undergone subtotal thyroidectomy at age 9 for hyperthyroidism [21]. In another family, two children and their mother were found to carry the same variant [22]. The children exhibited early-onset hyperthyroidism and goiter despite carbimazole therapy, while their mother had adolescent-onset hyperthyroidism treated with radioactive iodine ablation at age 24; the variant was identified retrospectively after the children's diagnoses [22]. An additional report described p.Ser505Asp substitution, also associated with congenital hyperthyroidism [45], reinforcing the functional importance of codon 505 in regulating receptor activation. Taken together, these cases illustrate the variable expressivity of the activating mutations at this site. However, none displayed the degree of early onset multiorgan involvement, including craniosynostosis, hydrocephalus, and mitral valve dysplasia, observed in our patient, highlighting the unique clinical severity of this de novo p.Ser505Arg variant.

The TSHR p. Asp633His variant in Case 2 has previously been observed in thyroid cancers and toxic nodules as well as murine model [25,46–46], but to our knowledge, this is the first report of a germline TSHR p.D633H variant in humans. This variant affects a critical hotspot region in TM6 of the TSHR, known to play a central role in maintaining receptor inactivity. Functional studies in COS-7 cells expressing D633H have shown constitutive activity for both the cAMP and the inositol phosphate signaling cascades [46, 47]. Studies into the highly conserved D633 residue demonstrate a critical interaction between transmembrane domains six and seven in stabilizing the inactive state of TSHR, suggesting that interruption of this bonding could lead to constitutive activity [46]. The pathogenic potential of this variant is further substantiated by TSHR D633H knock-in mouse model. In this model, homozygous mice developed overt hyperthyroidism by 2 months of age, and by 1 year, nearly all progressed to papillary thyroid carcinomas (PTCs) despite lacking mutations in common oncogenes [48]. Notably, heterozygous mice also developed thyroid carcinoma at a later age, highlighting the importance of long-term surveillance, even in patients with monoallelic variants. Follow-up studies revealed a potential link between TSHR signaling and the MAPK pathway in the generation of PTCs in D633H mice [49]. Other substitutions at this site—including p.Asp633Glu and p.Asp633Tyr—have also been reported in patients with nonautoimmune congenital or sporadic hyperthyroidism [50], further supporting the functional sensitivity and disease relevance of this residue. Collectively, these molecular and clinical findings establish codon 633 as a structurally and functionally critical determinant of TSHR activity, with mutations resulting in varying degrees of constitutive signaling, clinical severity, and potential for neoplastic transformation.

## 5. Conclusion

These two cases illustrate the severe clinical manifestations of SNAH caused by de novo TSHR activating variants, p.Ser505Arg and p.Asp633His. Case 1 expands the phenotype of TSHR p.S505R to include craniosynostosis and mitral valve dysplasia, not previously reported. Case 2 is the first known human case of a germline TSHR p.D633H variant, demonstrating profound thyroid hypertrophy and craniosynostosis. Together, these cases underscore the need for early genetic diagnosis and multidisciplinary management in infants with atypical features of hyperthyroidism.

## Data Availability

The data that support the findings of this study are available upon request from the corresponding author. The data are not publicly available due to privacy or ethical restrictions.
